# Well‐being in German patients with vitiligo in genital and visible areas – a pilot study

**DOI:** 10.1111/ddg.15891

**Published:** 2025-10-08

**Authors:** Janne Ohlenbusch, Rachel Sommer, Kerstin Steinbrink, Markus Böhm

**Affiliations:** ^1^ Department of Dermatology University of Münster Münster Germany; ^2^ Institute for Health Services Research in Dermatology and Nursing University Medical Center Hamburg‐Eppendorf Hamburg Germany

**Keywords:** Burden of disease, psychosocial impact, quality of life, questionnaire study

## Abstract

**Background:**

Only a few studies exist on the psychosocial impact of vitiligo in German patients, in particular those affected in genital and visible body areas.

**Methods:**

This monocentric pilot study aimed to assess well‐being of adult patients with vitiligo and to compare sex, age, and clinical characteristics between patients with and without genital, facial and hand involvement. Well‐being was assessed using the WHO‐5 questionnaire and quality of life with the Dermatology Life Quality Index (DLQI). Furthermore, psychometric properties of the WHO‐5 well‐being index were tested.

**Results:**

In total, 110 patients (mean age ± SD: 47.5 ± 14.7 years; 54.5% female; 95.5% with non‐segmental vitiligo) were included. The WHO‐5 mean score was 13.3, with 46 patients (42.2%) having a score < 13, indicating reduced well‐being. Sixteen patients (14.7%) had a WHO‐5 score < 7, indicating a high probability of depression. No significant differences in WHO‐5 or DLQI scores were found between patients with vs without genital, facial, and hand involvement. Psychometric properties were confirmed to be good.

**Conclusions:**

Our findings emphasize the psychosocial impact of vitiligo in German patients, as reflected by reduced well‐being, and suggest the usefulness of the WHO‐5 as a simple psychosocial screening tool for routine care.

## INTRODUCTION

Vitiligo is a common skin disorder with an estimated global prevalence of 1.1% (confirmed diagnosis) and 1.3% (unconfirmed diagnosis) based on a recent questionnaire survey study in the USA, Europe (including Germany), and Japan.^1^ The disease is caused by autoimmune‐mediated destruction of melanocytes resulting in white spots. Non segmental vitiligo (NSV) affecting the skin in a symmetric fashion is the most common subtype and often affects visible areas such as the face, neck, and dorsal aspects of hands and fingers.^2^ Surprisingly, there are only limited epidemiologic data on the psychosocial impact of vitiligo in German patients as emphasized in a recent position paper.^3^ Distribution of lesions in visible skin areas and female gender have previously been linked to stigmatization, as measured by the *Questionnaire on Experience with Skin Complaints* in German patients with vitiligo.^4^ In the most extensive study, published in 2009 and involving 3,319 patients from two German vitiligo self‐help associations, the mean value of the *Dermatology Life Quality Index* (DLQI), the most commonly used *Patient‐Reported Outcome Measure* (PROM) for chronic skin diseases, was 7.0 in 1,023 returned questionnaires.^5^ In light of accumulating evidence for impaired quality of life (QoL), disease burden, stigmatization, and psychosocial comorbidity in patients with vitiligo – as shown by numerous clinical studies worldwide^6^ – we emphasize the urgent need for new and additional studies on this topic in Germany, as well as for the routine assessment of psychosocial comorbidity.^3^ Regarding treatment options, patients with vitiligo limited to the facial area are eligible for the Janus kinase (JAK) 1/2 inhibitor ruxolitinib as the first officially approved topical therapy. However, genital and hand lesions might be associated with a severe impairment in QoL and well‐being, as well. Unfortunately, genital lesions often remain undisclosed because of embarrassment and discomfort about discussing sex‐related issues during healthcare visits. Here, we present data from a cross‐sectional, monocentric pilot study on the impact of vitiligo on health‐related well‐being in general and, in comparison, between patients with and without genital and visible (facial and hand) involvement. In addition, psychometric properties of the *WHO‐5 questionnaire for vitiligo* were tested.

## MATERIAL AND METHODS

### Study Design

This was a cross‐sectional pilot study under routine conditions in patients with vitiligo aged 18 years and older. Approval was obtained from the Ethical Committee Westfalen‐Lippe of the Ärztekammer Westfalen‐Lippe (2022‐384‐f‐S).

### Patients

Adult patients with an established dermatologic diagnosis of vitiligo undergoing routine care at the Outpatients Department of the Department of Dermatology, University of Münster, Germany, were retrieved from the electronic patient chart data system (Orbis) between January 2018 and November 2023 by utilizing the report generator and the *International Statistical Classification of Diseases and Related Health Problems Code L80.0*. This included patients covered only by public health insurance, but not those covered by private health insurance or patients hospitalized for other diagnoses. Only patients with precisely classified vitiligo (NSV, segmental vitiligo [SV], or undetermined), a complete medical history, and documentation of affected body sites were included. The selected patients were then sent the generic questionnaires listed below, along with an information and consent letter. All individuals were provided with a third standardized questionnaire for self‐assessment of their skin phototype. The latter questionnaire is freely available from the Bundesamt für Strahlenschutz (https://www.bfs.de/DE/themen/opt/uv/wirkung/hauttypen/hauttypen.html). Data analysis was performed after pseudonymization.

### Outcome parameters

Two standardized questionnaires were completed by the patient:

‐ The German version of the WHO‐5.^7^ The WHO‐5 is a generic global rating scale measuring subjective well‐being containing positive statements (https://www.who.int/publications/m/item/WHO‐UCN‐MSD‐MHE‐2024.01). The respondent is asked to rate how well each of the five items applies to him or her when considering the last 14 days. Each of the five items is rated from 5 (all of the time) to 0 (none of the time). The raw score therefore theoretically ranges from 0 (absence of well‐being) to 25 (maximal well‐being).
‐ The DLQI.^8^ The DLQI is a skin‐generic QoL questionnaire that includes ten items to be answered in a 4‐point Likert response scale ranging from 0 (not at all) to 3 (very much). Eight out of ten items also include a “not relevant” response option, which was also scored as zero. A total sum score ranging from 0 to 30 was computed, with higher scores indicating greater impairment. DLQI scores > 10 were considered major/extreme impairments on patients’ lives.^9^
‐ Besides, age and sex, clinical variables (i.e., type of vitiligo, body surface area [BSA], disease duration, body area affected and comorbidities) were obtained from the patient´s electronic chart and medical report. These variables were generated by the treating dermatologist. Selection of the skin phototype was made by the patient with the questionnaire as mentioned above.

### Statistical analysis

Data were analyzed using SPSS (Statistical Package für Social Sciences, Version 28), and Microsoft Excel (Version 16.87), assuming the critical p value (α) = 0.05 as the level of significance. Descriptive statistics (absolute [n] and relative frequencies [%] for categorical variables; mean and standard deviations [M ± SD] for continuous variables) were obtained for age, sex, and clinical variables as well as for the WHO‐5 and the DLQI. The homogeneity of sample age, sex, and clinical characteristics between the groups of patients with and without genital lesions was examined by independent samples t‐tests (continuous variables) or chi‐squared tests (categorical variables). To compare the QoL and well‐being of genital vs. non‐genital, facial vs. non‐facial and hand vs. non‐hand involvement, one‐way univariate analyses of covariance (ANCOVA) were performed for the DLQI and WHO‐5, including age, sex, BSA, comorbidities, and facial involvement (for genital comparison only) as covariates.^10^ Effect sizes were presented for the comparative analyses, considering ŋ^2^_p_ ≥ 0.01, ŋ^2^_p_ ≥ 0.06, and ŋ^2^_p_ ≥ 0.14 as small, medium, and large effects, respectively.^11^ For psychometric validation purposes, item distribution characteristics such as mean (M), standard deviation (SD), percentage of items at the lower (floor effects) and the higher end (ceiling effects), skewness were analyzed. For internal consistency Cronbach's alpha was calculated. Scores above 0.70 were interpreted as acceptable.^12^ To test for convergent validity, the WHO‐5 was correlated with the DLQI (Pearson's Correlation Coefficient). In line with Weber & Lamb (1970),^13^ scores between r = 0.36 and r = 0.67 were interpreted as moderate. Scores between r = 0.68 to r = 0.90 indicate high correlations and scores above 0.90 indicate very high correlations.

## RESULTS

### Patient's age, sex, and clinical characteristics

From a total number of n = 293 individuals to whom the questionnaire was sent,  n = 110 returned the questionnaire. The full demographics of the sample population are shown in Table 1. The majority of patients had NSV (95.5%; n = 105). 54.5% of the patient cohort were females. The mean age of the population was 47.45 (SD = 14.65, median = 49, range 18–84 years) and the majority of patients (60.0%, n = 66) had a skin phototype III. 84.5% (n =  93) had an involvement of the face and 72.7% (n = 80) of the hands. Involvement of genital area was noted in 47.3% (n = 52). 78.2% of patients had concomitant involvement of genital and visible areas. In 67.3% (n = 74) BSA documentation was available. The mean extent of the disease in patients with documented BSA was 13.59% (SD: 17.10). 45.87% (n = 50) of patients had extensive vitiligo with a BSA > 6.45%. The mean disease duration was 12.36 years (SD = 11.62, median = 7.5, range: 1–48). The groups of patients with and without genital involvement were mainly homogeneous. However, in terms of age and sex, male patients were more often diagnosed with genital vitiligo than female patients. In addition, patients without genital involvement had more often recorded comorbidities than patients with genital involvement, and patients with genital involvement also had facial involvement significantly more often.

**TABLE 1:** Age, sex, and clinical characterization of patients with vitiligo with and without genital involvement.

		Total	Genital involvement	No genital involvement	X^2^/ t	p
***Age and sex (n)***		*110*				
Sex, n (%)	Male	50 (45.5)	29 (55.8%)	21 (36.2%)	4.23	0.055
	Female	60 (54.5)	23 (44.2%)	37 (63.8%)		
Age, M ± SD		47.45 (14.65)	48.00 ± 13.40	46.97 ± 15.79	0.368	0.713
***Disease characteristics***						
Type of vitiligo, n (%)	Non‐segmental vitiligo	105 (95.5)	49 (94.2%)	56 (96.6%)	0.34	0.666
	Segmental vitiligo	5 (4.5)	3 (5.8%)	2 (3.4%)		
%BSA, M ± SD		13.59 (17.10)	13.00 ± 19.47	14.10 ± 15.03	−0.274	0.785
	Missing, n (%)	36 (32.7)	18 (34.6%)	18 (31.0%)		
Disease duration (years), M ± SD		12.36 (11.62)	12.56 ± 11.17	12.14 ± 12.13	0.133	0.895
	Missing, n (%)	14 (12.7)	8 (50.0%)	8 (50.0%)		
Skin phototype, n (%)	Type I	0 (0%)	0 (0%)	0 (0%)	1.93	0.381
	Type II	35 (31.8)	16 (30.8%)	19 (32.8%)		
	Type III	66 (60.0)	29 (55.86%)	37 (63.8%)		
	Type IV	7 (6.4)	5 (9.6%)	2 (3.4%)		
	Type V	0 (0%)	0 (0%)	0 (0%)		
	Type VI	0 (0%)	0 (0%)	0 (0%)		
	Missing	2 (1.8)	2 (3.8)			
Comorbidities, n (%) yes/		46 (41.8)	15 (28.8%)	31 (53.4%)	6.82	0.012
no		64 (58.2)	37 (71.2%)	27 (46.6%)		
Sites of involvement	Face (yes), n (%)	93 (84.5)	49 (52.6%)	44 (47.4%)	7.080	0.009
	Hands (yes), n (%)	80 (72.7)	37 (46.3%)	43 (53.8%)	0.123	0.831

*Abbr*.: M, mean; SD, standard deviation; n, number; BSA, body surface area

### Patient‐reported outcomes on quality of life and well‐being

The mean WHO‐5 score was 13.29 (SD = 5.40) and mean DLQI score 7.40 (SD = 6.33), indicating greater impairment in well‐being than in QoL (Tables 2, 3). Descriptive statistics for WHO‐5 and DLQI are shown in Tables 3, 4. Preliminary correlation analyses showed that the WHO‐5 correlated negatively with age (r = –0.281; p = 0.003). In addition, DLQI correlated moderately with age (r = 0.402; p ≤ 0.001) and BSA (r = 0.312; 0.003).

**TABLE 2:** Comparative analyses (ANCOVA) of well‐being and quality of life between patients with and without genital, facial, and hand involvement.

	Genital involvement		No genital involvement					
	*n*	*M ± SD*	*n*	*M ± SD*	*F*	*df*	*p*	*ŋ^2^_p_*
QoL impairments (DLQI)	33	7.00 ± 5.45	40	7.48 ± 5.65	0.242	1 66	0.624	0.004
Well‐being (WHO‐5)	52	13.02 ± 5.39	58	13.59 ± 5.41	0.481	1 104	0.490	0.005

	Facial involvement		No facial involvement					
	*n*	*M ± SD*	*n*	*M ± SD*	*F*	*df*	*p*	*ŋ^2^_p_*
QoL impairments (DLQI)	62	7.45 ± 5.67	11	6.18 ± 4.90	1.406	1 66	0.270	0.021
Well‐being (WHO‐5)	93	13.23 ± 5.29	17	13.82 ± 6.02	0.277	1 104	0.600	0.003

	Hand involvement		No hand involvement					
	*n*	*M ± SD*	*n*	*M ± SD*	*F*	*df*	*p*	*ŋ^2^_p_*
QoL impairments (DLQI)	52	7.63 ± 6.19	21	6.80 ± 6.75	0.344	1 67	0.559	0.005
Well‐being (WHO‐5)	80	13.11 ± 5.13	30	13.87 ± 6.07	0.116	1 105	0.734	0.001

*Abbr*.: QoL, quality of life; DLQI, Dermatology Life Quality Index (range 0–30); WHO‐5, Well‐being Index (range 0–15); SD, standard deviation; ηp^2^, partial eta squared

*Note*: Effect sizes are presented for the comparative analyses, considering ηp^2^ ≥ 0.01, ηp^2^ ≥ 0.06, and ηp^2^ ≥ 0.14 as small, medium, and large effects, respectively.

**TABLE 3:** Descriptive statistics of WHO‐5 and DLQI.

	WHO‐5	Item 1:	Item 2:	Item 3:	Item 4:	Item 5:	DLQI
	Total Score Baseline (0–25)	Cheerful and in good spirit	Calm and relaxed	Active and vigorous	Fresh and rested	Interested	Total Score (0–30)
Mean	13.3	2.9	2.6	2.6	2.4	2.9	7.4
SD	5.4	1.2	1.4	1.2	1.4	1.2	6.3
Skewness	−0.3	−0.4	−0.3	−0.2	−0.1	−0.4	1.0
Kurtosis	−0.7	−0.8	−0.9	−0.7	−0.9	−0.7	0.5
Min	2	0	0	0	0	0	0
Max	25	5	5	5	5	5	27

WHO‐5 (0 = absence of well‐being to 25 = maximal well‐being); DLQI (0 = minimum impairment to 30 = maximum impairment).

*Abbr*.: SD, standard deviation

**TABLE 4:** Distribution characteristics of WHO‐5.

	WHO‐5 Item 1	WHO‐5 Item 2	WHO‐5 Item3	WHO‐5 Item 4	WHO‐5 Item 5
At no time	3 (2.8%)	8 (7.3%)	5 (4.6%)	13 (11.9%)	2 (1.8%)
Some of the time	16 (14.7%)	21 (19.3%)	16 (14.7%)	20 (18.3%)	18 (16.5%)
Less than half of the time	22 (20.2%)	15 (13.8%)	31 (28.4%)	20 (18.3%)	15 (13.8%)
More than half of the time	25 (22.9%)	34 (31.2%)	28 (25.7%)	29 (26.6%)	36 (33.0%)
Most of the time	39 (35.8%)	26 (23.9%)	27 (24.8%)	22 (20.2%)	31 (28.4%)
All of the time	4 (3.6%)	5 (4.5%)	2 (1.8%)	2 (4.7%)	7 (6.5%)

WHO‐5 (0 = absence of well‐being to 25 = maximal well‐being).

**FIGURE 1:**
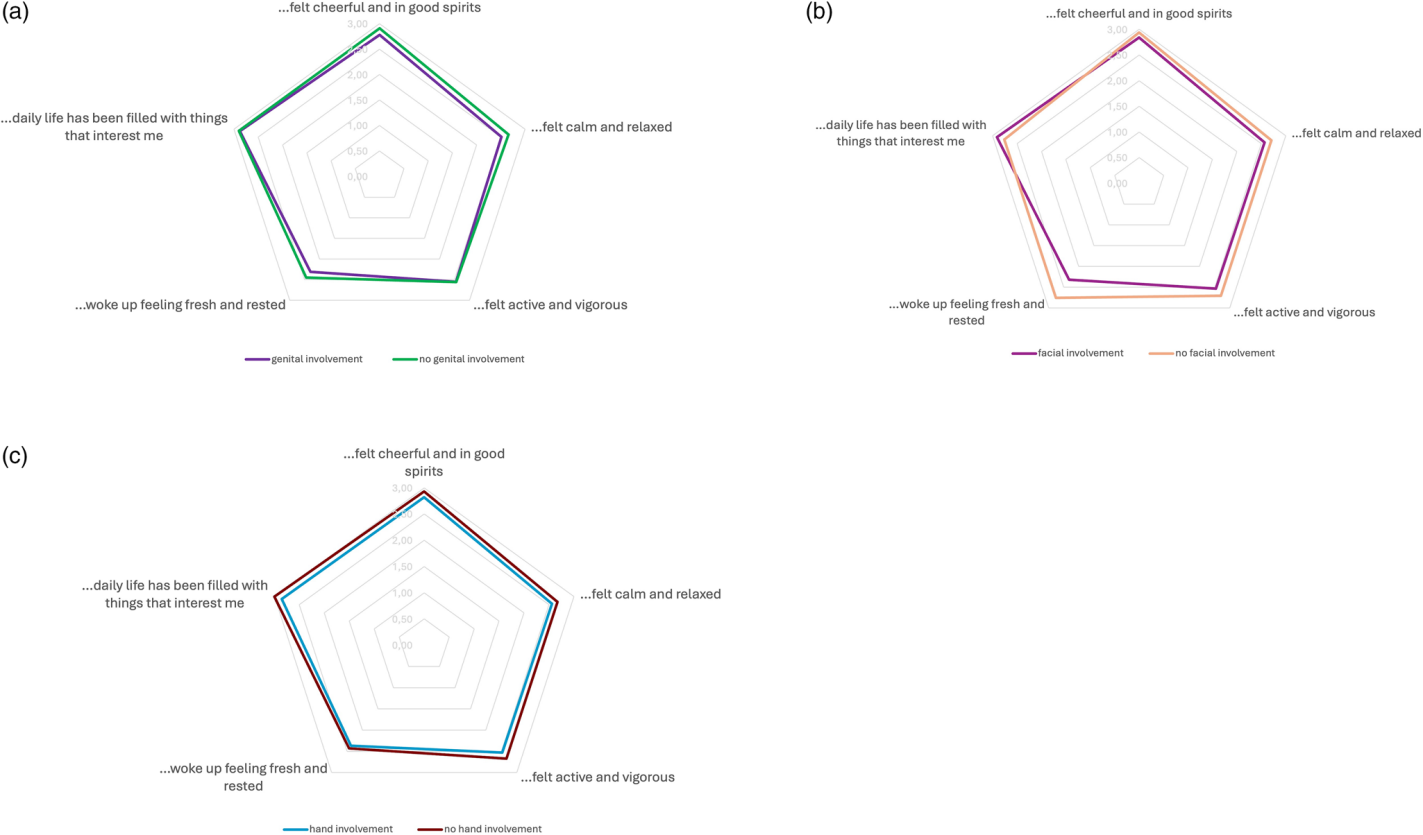
WHO‐5 items by genital, facial, and hand involvement (yes/no).

Comparative analyses showed no significant differences in the WHO‐5 and DLQI total score between patient with and without genital, facial, and hand involvement (Table 2). In line with this, WHO‐5 items show a similar pattern between the groups (Figure 1).

### Psychometric properties

The WHO‐5 showed good reliability, with Cronbach's alpha > 0.8 (95% CI 0.8–0.9), confirming internal consistency. Significant correlations with another patient‐reported outcome measure, namely the DLQI was found r = –0.408 (*p* < 0.01), confirming convergent validity.

## DISCUSSION

In this pilot study, we addressed health‐related well‐being, as measured by the WHO‐5, in German patients with vitiligo for the first time to the best of our knowledge. There is overwhelming global evidence that vitiligo is associated with a significant disease burden, as highlighted by numerous studies on QoL impairment.^3^, ^6^

In the majority of these surveys, whether in investigator‐ and pharmaceutical industry‐initiated studies or in clinical trials, the DLQI was utilized.^14^ However, even in the most recent phase III clinical trials leading to approval of topical ruxolitinib as the first officially approved treatment of patients with NSV,^15^ the DLQI failed to show significant changes of QoL during treatment in contrast to other PROMs, e.g. the vitiligo noticeability scale.^14^ This may be due to the lack of specificity of the DLQI compared to the VitiQoL.^16^ However, the VitiQoL is currently not freely available in the German language, hampering its usefulness in routine dermatology. There is currently no consensus on a stepwise approach for the variety of PROMs in vitiligo, for example with a clear‐cut algorithm to assess stigmatization, well‐being, QoL impairment, disease burden, disease severity and impact, and, more specifically, psychosocial comorbidity such as depression and anxiety disorders. Routine use of several PROMs per patient may be informative, but may also be partially redundant, time‐consuming, and without consequence if no decision is made on the subsequent interventional steps needed within the framework of a holistic therapeutic management for vitiligo. The position statement of the *International Task Force Vitiligo* proposed the use of the VitiQoL.^17^ We have recently highlighted the urgent need for screening of psychosocial disturbances and comorbidity in patients with vitiligo in an expert consensus paper.^3^ Indeed, the WHO‐5 is not vitiligo‐specific. However, it is less time‐consuming than the DLQI (5 versus 10 questions) and may serve as a freely available and easy to use initial screening tool for any patient with vitiligo before more specific tools such as the *Patient Health Questionnaire‐2* (PHQ‐2)^18^ or the *Generalized Anxiety Disorder 2‐item* (GAD‐2)^19^ are used. Together with the Professional Association of German Dermatologists (Berufsverband Deutscher Dermatologen [BVDD]), we proposed the WHO‐5 as a possible PROM in a checklist of documentation useful for dermatologists treating patients with vitiligo prior to prescribing Janus kinase inhibitors.^20^

Albeit a monocentric pilot study with a limited number of patients, the distribution of the subtypes of vitiligo (NSV vs. SV) and gender ratio in this study are in agreement with previous reports.^2^, ^21^ Accordingly, 5–16% of total vitiligo cases are segmental.^22^, ^23^ The mean age of our patient cohort, however, was higher than the reported peak incidence of vitiligo at 34 years in adults.^24^ As social factors, for example marital status, have been reported to affect QoL impairment in at least some studies,^14^ we can expect actual well‐being to be even lower in a more representative patient cohort. Notwithstanding these limitations, our study revealed that 14.68% (n = 16) of our patients have WHO‐5 scores of < 7, indicating a high probability of clinical depression. No recent data are currently available on the prevalence of depression in patients with vitiligo in Germany. Depending on the methodology used, the global frequency of depressions and depressive disorders ranged from 0.1–62.3%.^3^, ^6^

The WHO‐5 mean score was 13.3, indicating impairments in well‐being in vitiligo comparable to those in other chronic skin diseases such as psoriasis (M = 15.6),^25^ other chronic conditions such as type 2 diabetes (M = 12.6),^26^ and tumor diagnoses such as breast cancer (M = 13.3).^27^ Our pilot study did not reveal any correlation between impaired well‐being and the skin sites affected by vitiligo, for example visible areas such as the face or hands versus non‐visible areas such as the genital region. Most patients had involvement of the face and hands. A possible reason for this could be the overall low number of patients in our cohort who did not have involvement of the face or hands. Furthermore, previous studies have not shown a consistent impact of genital area involvement on QoL in patients with vitiligo.^14^ Likewise, neither darker skin phototype nor disease extent was statistically correlated with greater impairment of well‐being in our patients with vitiligo, most likely due to the low number of patients with skin phototypes higher than III (6.42%, n = 7). Interestingly, no significant differences in WHO‐5 and DLQI total scores were found for genital vs non‐genital, facial vs non‐facial, or hand vs non‐hand involvement, indicating that the impairments in well‐being and QoL are independent of the affected body area. However, except for the DLQI in patients with facial vs non‐facial involvement, all patients with facial, genital, or hand involvement reported lower well‐being and greater QoL impairment than patients without such involvement, although this was not statistically significant. Our results may be limited in terms of power due to the small sample size in subgroups. Thus, the association between specific visible (face, hands) or genital skin areas and well‐being must be investigated in larger, and ideally longitudinal, studies.

The secondary aim of the present study was to investigate validation features of the WHO‐5 questionnaire for use in people with vitiligo. According to the WHO definition, the essential characteristic of health is a state of complete well‐being. To meet such goals, a validated instrument measuring health‐related well‐being needs to be used. Health care studies in many indications show that the WHO‐5 questionnaire has favorable properties in terms of validity and feasibility. The WHO‐5 has proven to be valid in studies on the elderly population, for mental illnesses, diabetes, and neurological conditions.^28^, ^29^, ^30^, ^31^ In particular, validity has been demonstrated in depression screening, and a large spectrum of use in medicine has been revealed in a systematic review. Because of its small number of questions and its simple scoring method, it is particularly suitable for routine care.

The first dermatological use of the WHO‐5 was reported in acne vulgaris in the early 1980s, related to its potential function as a screening tool for depression.^32^ More recently, the psychometric properties of the WHO‐5 were confirmed in psoriasis.^25^ Our pilot study demonstrated positive properties for the use of the WHO‐5 in patients with vitiligo. The results suggest that the WHO‐5 can contribute to the assessment of patient well‐being in vitiligo routine care since the questionnaire showed good reliability and validity.

However, our study has limitations and weaknesses. First, the number of patients responding to the questionnaire was limited and included only patients treated in one clinical center in Germany. Secondly, the inclusion and exclusion criteria (see section Material and Methods) may have created a bias. Further, the age, sex, and clinical characteristics of patients who did not respond to our questionnaire could differ, as could their DLQI and WHO‐5 scores. Studying the impact of vitiligo on well‐being in larger cohorts of German patients using core outcome sets, as suggested by the Vitiligo International Task Force for an Agreed List of Core Data (VITAL),^33^ may confirm and extend our findings. Future studies should also investigate the responsiveness of well‐being in patients with vitiligo to treatment.

## CONFLICT OF INTEREST STATEMENT

M.B. has received honoraria from AbbVie, CME‐Welt, Incyte, MSD, and Pfizer (companies involved in developing medications for vitiligo) for advisory functions. He has also received honoraria from AbbVie, CME‐Welt, derCampus, Incyte, Isispharma, Janssen‐Cilag, Omnicuris, Pfizer, and StreamedUp for scientific presentations on vitiligo. He is subinvestigator or deputy principal investigator in clinical trials from AbbVie, Incyte, MSD, and Pfizer on patients with vitiligo. He is the recipient of an investigator‐initiated research grant from Incyte (VitiligoHealth/EU‐DE‐D‐24053). R.S. has received lecture, research, and consulting fees and/or travel expenses from AbbVie, Almirall, Amgen, Beiersdorf AG, CME‐Welt, EPG‐Health, Incyte, Janssen‐Cilag, Leo Pharma, Novartis, and UCB. K.S. and J.O. have no conflicts of interest.
